# Serotonin Transporter Imaging in Multiple System Atrophy and Parkinson's Disease

**DOI:** 10.1002/mds.29220

**Published:** 2022-09-14

**Authors:** Kelvin L. Chou, Praveen Dayalu, Robert A. Koeppe, Sid Gilman, C. Chauncey Spears, Roger L. Albin, Vikas Kotagal

**Affiliations:** ^1^ Department of Neurology University of Michigan Ann Arbor Michigan USA; ^2^ University of Michigan Udall Center Ann Arbor Michigan USA; ^3^ Division of Nuclear Medicine, Department of Radiology University of Michigan Ann Arbor Michigan USA; ^4^ Veterans Affairs Ann Arbor Health System (VAAAHS) and VAAAHS Geriatric Research Education and Clinical Center Ann Arbor Michigan USA; ^5^ University of Michigan Parkinson's Foundation Research Center of Excellence Ann Arbor Michigan USA

**Keywords:** multiple system atrophy, serotonin, [^11^C]DASB, PET imaging, Parkinson disease

## Abstract

**Background:**

Both Parkinson's disease (PD) and multiple system atrophy (MSA) exhibit degeneration of brainstem serotoninergic nuclei, affecting multiple subcortical and cortical serotoninergic projections. In MSA, medullary serotoninergic neuron pathology is well documented, but serotonin system changes throughout the rest of the brain are less well characterized.

**Objectives:**

To use serotonin transporter [^11^C]3‐amino‐4‐(2‐dimethylaminomethyl‐phenylsulfaryl)‐benzonitrile positron emission tomography (PET) to compare serotoninergic innervation in patients with MSA and PD.

**Methods:**

We performed serotonin transporter PET imaging in 18 patients with MSA, 23 patients with PD, and 16 healthy controls to explore differences in brainstem, subcortical, and cortical regions of interest.

**Results:**

Patients with MSA showed lower serotonin transporter distribution volume ratios compared with patients with PD in the medulla, raphe pontis, ventral striatum, limbic cortex, and thalamic regions, but no differences in the dorsal striatal, ventral anterior cingulate, or total cortical regions. Controls showed greater cortical serotonin transporter binding compared with PD or MSA groups but lower serotonin transporter binding in the striatum and other relevant basal ganglia regions. There were no regional differences in binding between patients with MSA–parkinsonian subtype (n = 8) and patients with MSA–cerebellar subtype (n = 10). Serotonin transporter distribution volume ratios in multiple different regions of interest showed an inverse correlation with the severity of Movement Disorders Society Unified Parkinson's Disease Rating Scale motor score in patients with MSA but not patients with PD.

**Conclusions:**

Brainstem and some forebrain subcortical region serotoninergic deficits are more severe in MSA compared with PD and show an MSA‐specific correlation with the severity of motor impairments. © 2022 The Authors. *Movement Disorders* published by Wiley Periodicals LLC on behalf of International Parkinson and Movement Disorder Society.

Dysfunction of caudal brainstem serotoninergic raphe nuclei is suggested to occur early in Parkinson's disease (PD).^1^ The relative frailty of these nuclei in PD may relate to their high baseline metabolic activity^2^ or their key role in interconnected brainstem projection system networks^3^ implicated in the cell‐to‐cell spread of misfolded synuclein. Multiple system atrophy (MSA) is a parkinsonian disorder with neuronal cell loss in several neurotransmitter projection systems.^4^ There are two clinical subtypes of MSA: a parkinsonian subtype (MSA‐P), presenting primarily with parkinsonism, and a cerebellar subtype (MSA‐C), presenting as a progressive cerebellar disorder with later expression of parkinsonism. MSA is characterized by glial cytoplasmic synuclein pathology rather than the neuron inclusions characteristic of PD.^5^ There is clear neuropathologic evidence of serotoninergic neuronal loss in the caudal brainstem raphe nucleus complex in MSA.^6^, ^7^ These neurons are known to project within the caudal brainstem and to motor and intermediolateral areas of the spinal cord. Their degeneration may contribute to MSA‐specific features, including respiratory and autonomic dysfunction.

We know less about the extent of rostral serotoninergic projection system degeneration in MSA compared with PD. Rostral serotoninergic projections to the striatum, thalamus, and limbic cortices are implicated in PD as risk factors for sleep difficulties,^8^ affective symptoms,^9^ and weight changes.^10^ Similar nonmotor features are common and severe in MSA,^11^ although their biological basis is less well understood. [^11^C]3‐amino‐4‐(2‐dimethylaminomethyl‐phenylsulfaryl)‐benzonitrile ([^11^C]DASB) is a positron emission tomography (PET) tracer that binds to the serotonin transporter (SERT) and can quantify regional serotoninergic projection terminal density,^12^, ^13^, ^14^ providing an opportunity to examine rostral serotoninergic projection system terminal integrity in patients with MSA compared with PD.

## 1 Methods

We conducted a single‐center, observational, cross‐sectional neuroimaging study to evaluate rostral brain [^11^C]DASB binding in MSA and compared these findings with existing DASB data available at the University of Michigan (UM) PET center from participants with PD. Patients were recruited from movement disorders clinics and through a web‐based posting on a UM human subjects recruitment website (https://umhealthresearch.org/). All patients signed informed consent documents approved by the UM Medical Institutional Review Board.

Patients with MSA were aged 30 to 80 years with a diagnosis of possible or probable MSA‐P or MSA‐C according to the MSA Second Consensus Criteria^15^ and had a Mini‐Mental State Examination score of 24 or greater. Patients with PD were aged 45 years or older with modified Hoehn and Yahr stages 1 to 4 and met the UK Brain Bank Clinical Diagnostic Criteria for PD.^16^ PET data on the patients with PD in this cohort has been reported previously.^17^ We excluded patients with contraindications to magnetic resonance imaging or PET imaging and those taking serotoninergic or anticholinergic medications in the 2 months preceding enrollment. For comparison to both PD and MSA, we included [^11^C]DASB data obtained from our PET center on a group of healthy controls (HCs).

All patients underwent [^11^C]DASB PET imaging. Methods for our group's [^11^C]DASB approach are detailed elsewhere.^17^, ^18^ Briefly, we injected patients with an intravenous bolus followed by an 80‐minute infusion of [^11^C]DASB. PET images were motion corrected and normalized into a common atlas space using NeuroStat software (https://neurostat.neuro.utah.edu). We defined volumes of interest (VOIs), including Brodmann areas and subcortical gray matter structures, on PET images using a Talairach brain atlas.^19^ These brain regions were identified on normalized parametric K_1_ PET images using a set of predefined Talairach atlas regions through the NeuroStat software package (additional details on this approach are described elsewhere^20^, ^21^). We used equilibrium modeling to estimate the distribution volume in each voxel. Distribution volume ratios (DVRs) were calculated as a mean for each set of paired right and left hemisphere VOIs. We similarly calculated the mean of right and left brainstem VOIs including the medulla, raphe pontis, dorsal raphe, and substantia nigra. Given the potential for the confounding influence of partial volume effects in the cerebellum in an MSA cohort, a disorder characterized by regional cerebellar atrophy, we used superior supratentorial white matter as a normalization reference region for calculating individual patients' [^11^C]DASB DVR. We selected this region because it had a relatively lower level of [^11^C]DASB binding, not differing significantly between patients with PD and MSA. Another PET imaging group used a similar approach and reported low reference region SERT binding.^22^

We explored differences in intergroup DVRs in several different VOIs. The medulla, raphe pontis, dorsal raphe, substantia nigra, ventral striatum, ventral anterior cingulate cortex (Brodmann Area 24), and thalamus were defined on atlas images. Distinct from the ventral striatum, we defined the dorsal striatum VOI as the voxel‐adjusted mean of the bilateral caudate nucleus and putamen. Limbic cortex was defined using the amygdala, hippocampus, and insula VOIs. The total cerebral cortex was defined using voxel‐adjusted volumes specific to relevant Brodmann areas and limbic regions identified on the NeuroStat atlas, and the thalamic VOI was identified through the Talairach atlas. We used descriptive statistics to display the means and proportions between the MSA, PD and HC groups. We used two‐sample *t* tests to compare patients with MSA and PD in each of these VOIs. We conducted exploratory analyses within PD and MSA groups to evaluate for bivariate correlations (Pearson's *r*) between regional [^11^C]DASB DVR and clinical scales, including the Movement Disorders Society Revised Unified Parkinson's Disease Rating Scale (MDS‐UPDRS) motor examination, the Montreal Cognitive Assessment (MoCA), the Geriatric Depression Scale (GDS), and the Epworth Sleepiness Scale (ESS).

## 2 Results

A total of 59 patients consented to participate in the study, but we excluded two patients from analysis (one patient with MSA who could not complete the [^11^C]DASB scan and one patient with MSA who was taking a serotonergic medication), leaving a total cohort of 57 (23 patients with PD, 18 patients with MSA, and 16 HCs). In the MSA group, there were 10 patients with MSA‐C and eight patients with MSA‐P. We found no intergroup differences in any of the regions of interest (ROIs) on [^11^C]DASB PET imaging between the two MSA subtypes, so they were subsequently combined into one group for comparison with PD (Table 1).

**TABLE 1:** Cohort characteristics and regional [^11^C]DASB DVRs

	PD, n = 23	MSA, n = 18	Healthy controls, n = 16	χ^2^/*t* test. *P* value comparing PD to MSA
Age, years	66.2 (6.7)	59.6 (7.6)	66.0 (5.2)	*t* = 2.932, *P* = 0.006
Sex	Female = 6, male = 17	Female = 8, male = 9	Female = 10, male = 6	χ^2^ = 1.89, *P* = 0.17
Disease duration, years	4.9 (4.1) [range, 1–12 years]	2.9 (2.8) [range, 1–11 years]	–	*t* = 1.713, *P* = 0.095
Modified HY scale	HY1 (n = 2), HY1.5 (n = 4), HY2 (n = 9), HY2.5 (n = 6), HY3 (n = 2)	–	–	
MDS‐UPDRS motor exam score	25.522 (9.050)	30.25 (19.033) [n = 14]	–	*t* = 1.023, *P* = 0.314
Montreal Cognitive Assessment score	25.565 (2.352)	24.333 (4.451) [n = 15]	–	*t* = 1.115, *P* = 0.272
Geriatric Depression Scale score	7.217 (4.512)	8.857 (4.504) [n = 14]	–	*t* = 1.073, *P* = 0.291
Epworth Sleepiness Scale score	6.826 (4.141)	6.571 (3.877) [n = 14]	–	*t* = 0.186, *P* = 0.854
Region of interest				
Total cortex	1.267 (0.130)	1.232 (0.093)	1.792 (0.136)	*t* = 0.964, *P* = 0.341
Caudate and putamen	2.232 (0.182)	2.210 (0.192)	2.007 (0.175)	*t* = 0.379, *P* = 0.707
Thalamus	2.381 (0.209)	2.187 (0.164)	2.103 (0.236)	*t* = 3.243, *P* = 0.002
Limbic cortex	1.553 (0.130)	1.454 (0.138)	1.799 (0.144)	*t* = 2.365, *P* = 0.023
Ventral striatum	2.388 (0.194)	2.205 (0.241)	1.959 (0.178)	*t* = 2.702, *P* = 0.010
Ventral anterior cingulate	1.384 (0.115)	1.360 (0.117)	1.650 (0.137)	*t* = 0.656, *P* = 0.516
Substantia nigra	2.416 (0.360)	2.401 (0.346)	1.614 (0.173)	*t* = 0.138, *P* = 0.891
Dorsal raphe	2.970 (0.342)	2.912 (0.390)	1.700 (0.139)	*t* = 0.509, *P* = 0.614
Raphe pontis	2.566 (0.297)	2.241 (0.300)	1.556 (0.253)	*t* = 3.464, *P* = 0.001
Medulla	1.571 (0.168)	1.364 (0.210)	1.464 (0.189)	*t* = 3.501, *P* = 0.001

Values are expressed as mean (standard deviation); absolute values of *t* test/χ^2^ are presented.

Abbreviations: [^11^C]DASB, [^11^C]3‐amino‐4‐(2‐dimethylaminomethyl‐phenylsulfaryl)‐benzonitrile; DVR, distribution volume ratio; PD, Parkinson's disease; MSA, multiple system atrophy; HY, Hoehn and Yahr; MDS‐UPDRS, Movement Disorders Society Revised Unified Parkinson's Disease Rating Scale.

**FIG 1:**
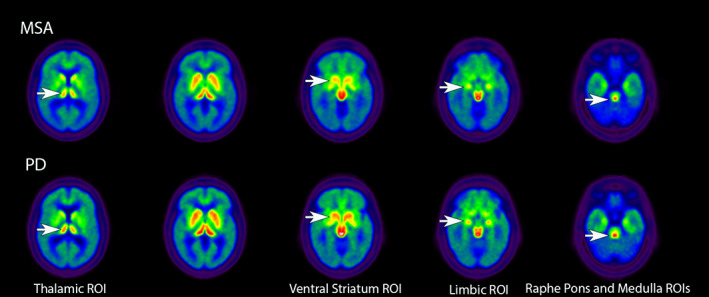
Axial images presenting group averages for [^11^C]3‐amino‐4‐(2‐dimethylaminomethyl‐phenylsulfaryl)‐benzonitrile (DASB) positron emission tomography in patients with MSA and PD. White arrows in the top rows show reduced [^11^C]DASB binding in MSA compared with PD in several ROIs. MSA, multiple system atrophy; PD, Parkinson's disease; ROI, region of interest.

Table 1 shows the differences between the groups in mean [^11^C]DASB DVRs in the selected ROIs. As expected, [^11^C]DASB binding in the cortical VOIs was higher in the HC group compared with patients with PD or MSA. Interestingly, the HCs showed a lower range of [^11^C]DASB binding in the striatum, ventral striatum, substantia nigra, dorsal raphe, and raphe pontis. There were no significant differences between patients with PD and MSA in total cortical, striatal, or substantia nigra [^11^C]DASB DVRs. Patients with MSA showed significantly lower [^11^C]DASB DVRs in the thalamus, limbic cortex, ventral striatum, raphe pontis, and medulla compared with patients with PD. Figure 1 depicts the [^11^C]DASB binding overlap between patients with MSA and PD in many supratentorial regions with qualitatively lower [^11^C]DASB binding in MSA in the limbic cortex and caudal brainstem. There were no sex differences seen in the PD group or in the MSA group in [^11^C]DASB DVR in any of the ROIs.

Patients with MSA and PD did not differ in MDS‐UPDRS motor exam, MoCA, GDS, or ESS scores (Table 1). Correlations between these clinical variables and regional [^11^C]DASB DVRs are presented in Tables S1 and S2. Given the potential for inflated type 1 error due to multiple comparisons, these bivariate correlations are intended to be interpreted as hypothesis generating in nature. For the most part, the MoCA, GDS, and ESS scores did not show a consistent correlative pattern with [^11^C]DASB DVR in either patients with PD or MSA. Interestingly, patients with MSA showed an inverse correlation between elevated MDS‐UPDRS scores and lower [^11^C]DASB DVRs seen in multiple different brain regions (Fig. S1). Outside of the striatum, these associations were not seen in patients with PD. Clinical data were available for only four of the eight total patients with MSA‐P, limiting our ability to conduct MSA subtype‐specific analyses. Even so, among the 10 patients with MSA‐C with available clinical data, similar patterns of inverse correlations were seen between MDS‐UPDRS motor score and [^11^C]DASB DVR, including in the total cortex (*r* = −0.7140, *P* = 0.0204), striatum (*r* = −0.7054, *P* = 0.0227), ventral striatum (*r* = −0.7457, *P* = 0.0133), and ventral anterior cingulate (*r* = −0.7930, *P* = 0.0062), but not in any of the other ROIs.

## 3 Discussion

Relative to the participants with PD, the participants with MSA showed comparable levels of [^11^C]DASB binding in neocortical and dorsal striatal ROIs but lower [^11^C]DASB DVRs in key subcortical regions, including the thalamus, ventral striatum, and limbic cortex as well as greater reductions in caudal brainstem [^11^C]DASB binding in the raphe pontis and medulla. Patients with MSA and PD showed lower cortical [^11^C]DASB binding compared with the HCs but higher striatal, midbrain, and pontine [^11^C]DASB binding. These MSA/PD region‐specific findings may reflect the presence of compensatory physiology in subcortical serotoninergic systems. Lower [^11^C]DASB binding in patients with MSA in various brain regions correlated with greater motor impairment on the MDS‐UPDRS motor exam. Our findings are from a relatively small cohort and may very well be impacted by multiple comparisons. For this important reason, they require replication in independent data sets. Nevertheless, these data comprise the first [^11^C]DASB study in MSA. Our findings confirm the presence of lower brainstem serotoninergic pathology in MSA and raise the possibility of relatively selective rostral serotoninergic network abnormalities as a distinctive pathological feature of MSA.

The rostral serotoninergic raphe complex, located in the midbrain/rostral pons, gives rise to two distinct groups of ascending fibers—the dorsal and ventral/median raphe pathways. The dorsal raphe projects to the striatum and globus pallidus, whereas the ventral/median raphe sends projections to other forebrain regions, including the thalamus, hypothalamus, and limbic structures. Previous neuropathological studies demonstrated advanced serotoninergic neurodegeneration in the medullary raphe complex of patients with MSA,^6^, ^7^, ^23^ a result consistent with our [^11^C]DASB PET findings. In contrast to the marked caudal brainstem serotoninergic neuron loss, a separate postmortem study showed only mild degeneration of the midbrain dorsal and median raphe complex in MSA, comprising an intermediate stage between HCs and more severe findings found in patients with Lewy body dementia.^24^ In this latter postmortem study, the patients with MSA had a mean disease duration of 7 years. Our study expands on these findings by characterizing serotoninergic system changes in participants with MSA with an average disease duration of only 3 years. The in vivo SERT density measurements of the cortex and dorsal striatum in our patients with MSA was similar to PD and consistent with prior postmortem findings. Our finding of comparable in vivo serotonin transporter density in the cortex and dorsal striatum in patients with MSA and PD could support the conclusion that abnormalities of some key serotoninergic projections are similar in PD and MSA. We found significant differences in some regions, suggesting differential loss of serotoninergic terminals and perikarya. Alternatively, SERT expression might be differentially regulated in PD and MSA, possibly reflecting differential compensatory capacity of serotoninergic systems in these two disorders. Longitudinal studies using multiple serotoninergic tracers are needed to clarify what PET findings reflect degenerative terminal loss versus endogenous compensatory responses.

Interestingly, patients with MSA and PD showed lower cortical but higher basal ganglia, midbrain, and pontine [^11^C]DASB DVRs compared with controls. There are several possible explanations for these intergroup findings, including the possibility that the serotoninergic system may show unique compensatory features in synucleinopathies. Prange and colleagues recently reported increased [^11^C]DASB binding in the ventral striatum and anterior cingulate cortex of patients with PD with apathy, suggesting that regional [^11^C]DASB elevations may occur in the setting of local neurodegeneration.^25^ Another possibility is that rising SERT density is not simply a synaptic phenomenon but might also carry an autoregulatory role in the proximal cell bodies of serotoninergic projection neurons. An electrophysiologic study has shown that local application of selective serotonin reuptake inhibitors to the dorsal raphe nucleus of mice leads to local SERT inhibition at the level of the cell bodies and subsequent enhancement of synaptic serotonin release.^26^ A third possibility is that our use of the superior supratentorial white matter as a reference region may have affected intergroup comparisons. This approach has not been reported or studied previously for [^11^C]DASB. We chose this region rather than the cerebellar [^11^C]DASB reference region given the potential for cerebellar pathology in MSA to bias intergroup comparisons. It is worth noting, however, that supratentorial white matter pathology has been described in MSA compared with controls.^27^ As such, this region may not be free of bias. Prospective validation of this approach against a model dependent on arterial sampling will be needed moving forward.

These results showing elevated regional [^11^C]DASB binding in PD compared with controls in the rostral brainstem and basal ganglia contrast with those reported in other cohorts.^8^, ^28^ At least one PD study suggests that certain subcortical regions may show declines in [^11^C]DASB binding with advancing disease duration.^29^ Nevertheless, the story of how regional [^11^C]DASB binding changes in PD is more nuanced and does not fit a monotonic, unidirectional pattern. Paradoxical elevations in [^11^C]DASB binding in the hypothalamus and hippocampus compared with controls have been reported in PD,^30^ as have paradoxical [^11^C]DASB upregulations in patients with PD and depression in the amygdala, hypothalamus, caudal raphe nuclei, and posterior cingulate cortex.^31^ Differences in published [^11^C]DASB PD findings most likely reflect the complicated influence of baseline differences in serotoninergic integrity and genetic risk factors, variability across cohorts in disease duration, previous use of drugs relevant to the serotoninergic nervous system, and heterogeneity of clinical features known to correlate with serotoninergic pathology. A multicenter, PD observational study involving multiple serotoninergic tracers would be the optimal next step to better understanding the natural history of [^11^C]DASB bindings in synucleinopathies.

Our exploratory clinical correlative analyses showed an MSA‐specific inverse correlation between the severity of motor impairment and serotoninergic integrity in several different cortical, basal ganglia, and brainstem ROIs. This is a novel finding and suggests that serotoninergic degeneration may be a possible therapeutic target for motor progression in MSA. Our findings are potentially confounded by the possibility of type 1 error and should be interpreted cautiously.

In addition to its cross‐sectional design, another relevant limitation of our study is its lack of a uniform clinical assessment battery; the patients with MSA and PD were recruited and imaged through different clinical research protocols. MSA and PD are known to have overlapping clinical features but distinctive disease‐specific clinical factors possibly related to the serotoninergic system dysfunctions. We were also missing clinical data in four patients with MSA‐P. Patients with PD were on average 6.6 years older than patients with MSA and had a mean disease duration that was 2 years longer. These findings may very well have impacted the results of our study. For example, it is possible that the clinical correlations seen between MDS‐UPDRS scores and [^11^C]DASB DVRs in patients with MSA only reflect dynamic changes seen in early disease that reach a ceiling effect as disease duration advances. The higher overall MDS‐UPDRS scores seen in patients with MSA in our cohort though would argue slightly against the possibility that patients with MSA may have less advanced parkinsonism compared with patients with PD.

The potential of the ascending serotoninergic projection system as a biomarker or therapeutic target in MSA is yet to be determined. Nevertheless, given the paucity of current MSA treatments and the clinical availability of serotoninergic drugs targeting different 5HT receptor classes, understanding serotoninergic system alterations in MSA and other synucleinopathies may have diagnostic and therapeutic relevance. The present study is a step toward elucidating serotoninergic network dysfunction in this rare and complex disease.

## 4 Author Roles

(1) Research Project: A. Conception, B. Organization, C. Execution; (2) Statistical Analysis: A. Design, B. Execution, C. Review and Critique; (3) Manuscript: A. Writing of the First Draft, B. Review and Critique.

K.L.C.: 3A, 3B

P.D.: 1B, 1C, 2C, 3B

R.A.K.: 1B, 1C, 3B

S.G.: 1A, 1B, 1C, 3B

C.C.S.: 3B

R.L.A.: 2C, 3B

V.K.: 2A, 2B, 2C, 3A, 3B

## 5 Financial Disclosures

K.L.C. reports consulting with Abbott, Accordant, Amneal, Avion Pharmaceuticals, and Neurocrine; royalties from UpToDate and Springer Publishing; and grants from The Michael J. Fox Foundation (Parkinson's Progression Markers Initiative), National Institutes of Health (NIH) (NS107158), Neuraly, and the Parkinson Study Group [Registry for the Advancement of DBS in PD (RAD‐PD)]. P.D. reports grants from the Huntington Study Group, Neurocrine, UniQure, and Vaccinex. R.L.A. reports grants from the National Institutes of Health (NIH) P50NS123067, the Parkinson's Foundation, and the Farmer Family Foundation; compensation for participation in the Data Safety and Monitoring Boards for the Signal‐AD trial (Vaccinex), the M‐STAR trial (Biohaven), and the TANGO trial (Biogen). V.K. receives research funding from the NIH and the VA Healthcare system as well as from the Movement Disorders Society as an associate editor service to the journal *Movement Disorders*. R.A.K., S.G., and C.C.S. have nothing to report.

## Supporting information


**Figure S1.** Scatter plots depicting the inverse relationship in patients with multiple system atrophy (MSA) (n = 14) between Movement Disorders Society Unified Parkinson's Disease Rating Scale (MDS‐UPDRS) motor score and regional [^11^C]3‐amino‐4‐(2‐dimethylaminomethyl‐phenylsulfaryl)‐benzonitrile (DASB) distribution volume ratio (DVR) in the (A) total cortex (top left), (B) caudate and putamen (top right), (C) ventral anterior cingulate (bottom left), and (D) raphe pontis (bottom right).
**Table S1.** Bivariate Pearson's correlations (*r* and *P* value) between regional [^11^C]3‐amino‐4‐(2‐dimethylaminomethyl‐phenylsulfaryl)‐benzonitrile (DASB) distribution volume ratio and clinical scales in patients with Parkinson's disease.
**Table S2.** Bivariate Pearson's correlations (*r* and *P* value) between regional [^11^C]3‐amino‐4‐(2‐dimethylaminomethyl‐phenylsulfaryl)‐benzonitrile (DASB) distribution volume ratio and clinical scales in patients with multiple system atrophy.
**Table S3.** Comparison of regional [^11^C]3‐amino‐4‐(2‐dimethylaminomethyl‐phenylsulfaryl)‐benzonitrile (DASB) distribution volume ratio (DVR) between normal controls versus Parkinson's disease and normal controls versus multiple system atrophy.

## Data Availability

The data that support the findings of this study are available from the corresponding author upon reasonable request.
