# Supplementary material for: Serotonin Transporter Imaging in Multiple System Atrophy and Parkinson's Disease
Source: Mov Disord. 2022 Sep 14;37(11):2301–7. doi: 10.1002/mds.29220 (PMC9669145; doi:10.1002/mds.29220)

Supplementary Table 1: Bivariate Pearson’s correlations (r and p-value) between regional DASB distribution volume ratio and clinical scales in subjects with Parkinson disease.

|  | MDS-UPDRS Motor exam [n=23] | Montreal Cognitive Assessment [n=23] | Geriatric Depression Scale [n=23] | Epworth Sleepiness Scale [n=23] |
| --- | --- | --- | --- | --- |
| Total Cortex | r=-0.137, p=0.533 | r=-0.089, p=0.686 | r=-0.218, p=0.318 | r=0.138, p=0.530 |
| Caudate & Putamen | r=-0.424,  p=0.044 | r=0.086, p=0.697 | r=-0.069, p=0.753 | r=0.271, p=0.210 |
| Thalamus | r=-0.250, p=0.251 | r=-0.034, p=0.879 | r=-0.100, p=0.650 | r=0.354  p=0.097 |
| Limbic Cortex | r=-0.137,  p=0.533 | r=0.013, p=0.953 | r=-0.221, p=0.310 | r=0.163  p=0.456 |
| Ventral Striatum | r=-0.236  p=0.279 | r=0.149  p=0.499 | r=-0.048  p=0.828 | r=0.382  p=0.073 |
| Ventral Anterior Cingulate | r=-0.234, p=0.282 | r=0.050, p=0.822 | r=-0.261,  p=0.229 | r=0.140,  p=0.524 |
| Substantia Nigra | r=-0.300, p=0.165 | r= 0.094, p=0.670 | r=0.121, p=0.583 | r=0.586, p=0.003 |
| Dorsal Raphe | r=-0.261, p=0.229 | r=-0.041, p=0.854 | r=0.125, p=0.570 | r=0.452, p=0.030 |
| Raphe Pontis | r=-0.229, p=0.292 | r=0.120, p=0.586 | r= 0.011, p=0.962 | r=0.338, p=0.115 |
| Medulla | r=-0.097, p=0.660 | r=0.041, p=0.854 | r=-0.296, p=0.170 | r=0.287,  p=0.184 |

Supplementary Table 2: Bivariate Pearson’s correlations (r and p-value) between regional DASB distribution volume ratio and clinical scales in subjects with Multiple System Atrophy

|  | MDS-UPDRS Motor exam [n=14] | Montreal Cognitive Assessment [n=15] | Geriatric Depression Scale [n=14] | Epworth Sleepiness Scale [ n=14] |
| --- | --- | --- | --- | --- |
| Total Cortex | r=-0.670, p=0.009 | r= 0.196, p=0.485 | r=-0.450,  p=0.111 | r=-0.269, p=0.353 |
| Caudate & Putamen | r=-0.682, p=0.007 | r=0.213, p=0.445 | r=-0.429, p=0.126 | r=-0.124, p=0.673 |
| Thalamus | r=-0.527, p=0.053 | r=0.056, p=0.842 | r=-0.177, p=0.546 | r=-0.083,  p=0.777 |
| Limbic Cortex | r=-0.593  p=0.026 | r=0.063  p=0.823 | r=-0.450  p=0.107 | r=-0.190  p=0.515 |
| Ventral Striatum | r=-0.774  p=0.001 | r=0.199  p=0.476 | r=-0.383  p=0.177 | r=-0.265  p=0.359 |
| Ventral Anterior Cingulate | r=-0.789  p=0.001 | r= 0.376  p=0.167 | r=-0.408  p=0.147 | r=-0.342  p=0.232 |
| Substantia Nigra | r=-0.484  p=0.080 | r=-0.029  p=0.919 | r=-0.356  p= 0.211 | r=-0.508  p=0.064 |
| Dorsal Raphe | r=-0.380  p=0.180 | r=-0.066  p=0.814 | r=-0.498  p=0.070 | r=-0.033  p=0.912 |
| Raphe Pontis | r=-0.564  p=0.036 | r=0.007  p=0.979 | r=-0.537  p= 0.048 | r=-0.364  p=0.200 |
| Medulla | r=-0.524  p= 0.055 | r=0.180  p=0.522 | r=-0.318  p=0.269 | r=-0.534  p=0.049 |

Supplementary Table 3: Comparison of Regional DASB DVR between Normal Controls vs. Parkinson disease and Normal Controls vs. Multiple System Atrophy

| Region of Interest | Parkinson disease  (PD; n=23) | Multiple System Atrophy  (MSA; n=18) | Normal Controls (NC; n=16) | NC vs. PD (t-test, p-value) | NC vs. MSA (t-test, p-value) |
| --- | --- | --- | --- | --- | --- |
| Total Cortex | 1.267 (0.130) | 1.232 (0.093) | 1.792 (0.136) | t=12.205, p <0.001 | t=14.148, p <0.001 |
| Caudate & Putamen | 2.232 (0.182) | 2.210 (0.192) | 2.007 (0.175) | t=3.858, p<0.001 | t=3.201, p=0.003 |
| Thalamus | 2.381 (0.209) | 2.187 (0.164) | 2.103 (0.236) | t=3.887, p<0.001 | t=1.213, p=0.234 |
| Limbic Cortex | 1.553 (0.130) | 1.454 (0.138) | 1.799 (0.144) | t=5.571, p<0.001 | t=7.135, p<0.001 |
| Ventral Striatum | 2.388 (0.194) | 2.205 (0.241) | 1.959 (0.178) | t=7.016, p<0.001 | t=3.349, p=0.002 |
| Ventral Anterior Cingulate | 1.384 (0.115) | 1.360 (0.117) | 1.650 (0.137) | t=6.583, p<0.001 | t=6.664, p<0.001 |
| Substantia Nigra | 2.416 (0.360) | 2.401 (0.346) | 1.614 (0.173) | t=8.249, p<0.001 | t=8.224, p<0.001 |
| Dorsal Raphe | 2.970 (0.342) | 2.912 (0.390) | 1.700 (0.139) | t=14.035, p<0.001 | t=11.758, p<0.001 |
| Raphe Pontis | 2.566 (0.297) | 2.241 (0.300) | 1.556 (0.253) | t=11.089, p<0.001 | t=7.157, p<0.001 |
| Medulla | 1.571 ( 0.168) | 1.364 (0.210) | 1.464 (0.189) | t=1.859, p=0.071 | t=1.443, p=0.159 |

*NC = Normal Control; MSA = Multiple System Atrophy; PD = Parkinson disease; absolute values of t-test/chi-square are presented.

Supplementary Figure 1

Scatter plots depicting the inverse relationship in MSA subjects (n=14) between MDS-UPDRS motor score and regional DASB distribution volume ratio (DVR) in the A) Total Cortex [top left] B) Caudate & Putamen [top right] C) Ventral Anterior Cingulate [bottom left] and D) Raphe Pontis [bottom right]


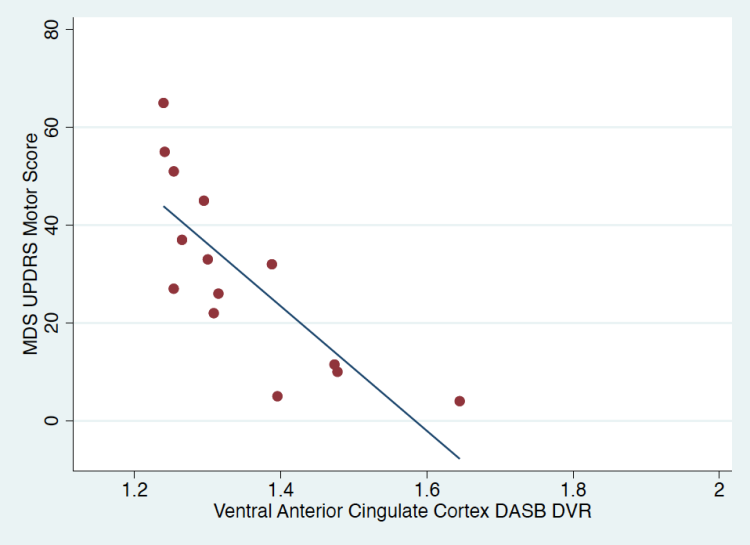

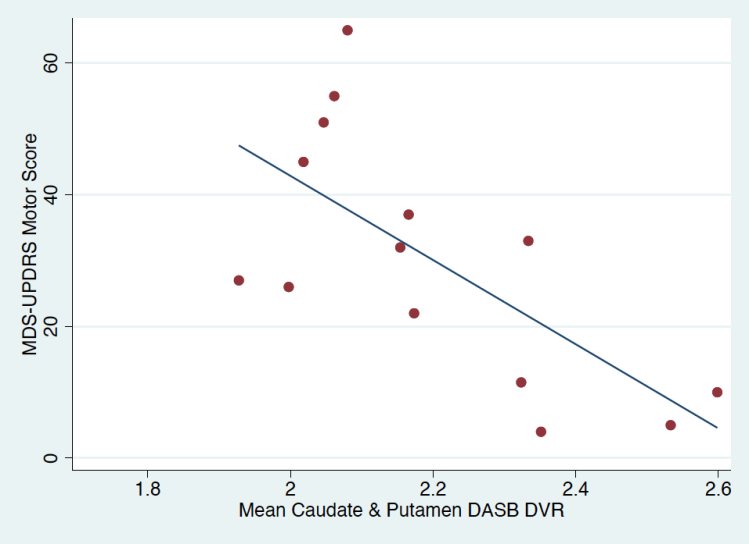


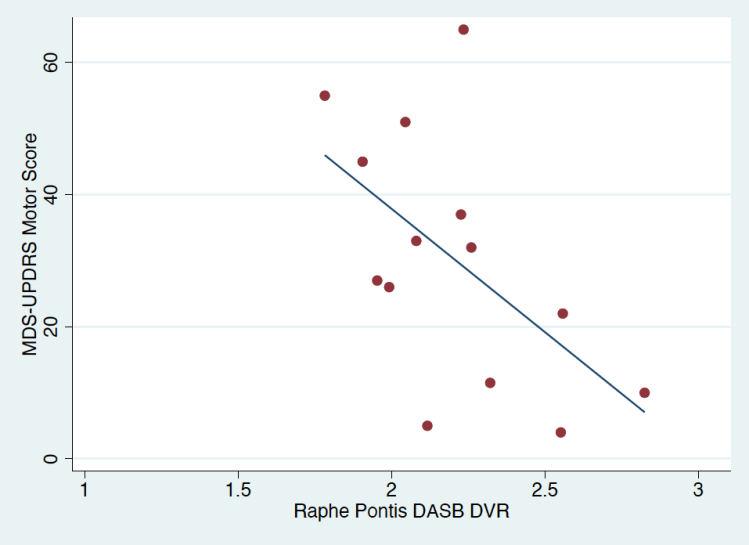

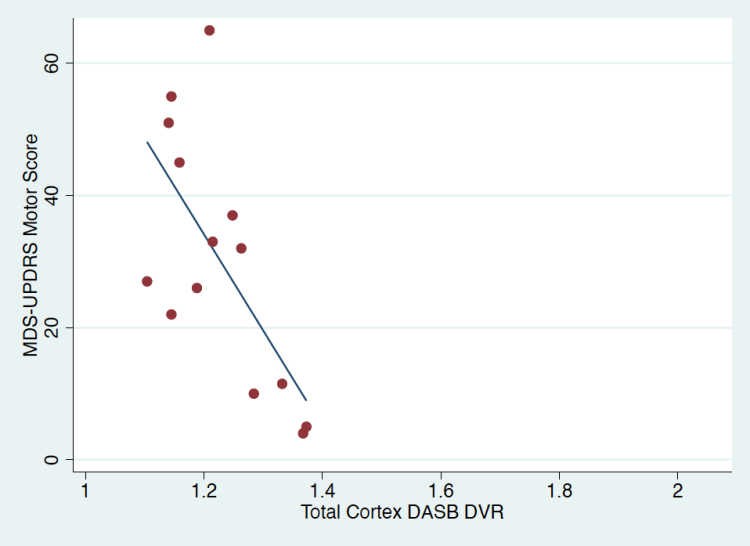

Supplement: Supplementary file 1 — Figure S1. Scatter plots depicting the inverse relationship in patients with multiple system atrophy (MSA) (n = 14) between Movement Disorders Society Unified Parkinson's Disease Rating Scale (MDS‐UPDRS) motor score and regional [11C]3‐amino‐4‐(2‐dimethylaminomethyl‐phenylsulfaryl)‐benzonitrile (DASB) distribution volume ratio (DVR) in the (A) total cortex (top left), (B) caudate and putamen (top right), (C) ventral anterior cingulate (bottom left), and (D) raphe pontis (bottom right). Table S1. Bivariate Pearson's correlations (r and P value) between regional [11C]3‐amino‐4‐(2‐dimethylaminomethyl‐phenylsulfaryl)‐benzonitrile (DASB) distribution volume ratio and clinical scales in patients with Parkinson's disease. Table S2. Bivariate Pearson's correlations (r and P value) between regional [11C]3‐amino‐4‐(2‐dimethylaminomethyl‐phenylsulfaryl)‐benzonitrile (DASB) distribution volume ratio and clinical scales in patients with multiple system atrophy. Table S3. Comparison of regional [11C]3‐amino‐4‐(2‐dimethylaminomethyl‐phenylsulfaryl)‐benzonitrile (DASB) distribution volume ratio (DVR) between normal controls versus Parkinson's disease and normal controls versus multiple system atrophy. [file MDS-37-2301-s001.docx]
