# Supplementary material for: Serotonin Transporter Imaging in Multiple System Atrophy and Parkinson’s Disease
Source: Mov Disord. Author manuscript; Available in PMC 2023 Nov 1. (PMC9669145; doi:10.1002/mds.29220)
Supplement: Supplementary file 1 [file NIHMS1832722-supplement-SUPINFO.docx]

Supplementary Table 1: Bivariate Pearson’s correlations (r and p-value) between regional DASB distribution volume ratio and clinical scales in subjects with Parkinson disease.

|  | MDS-UPDRS Motor exam [n=23] | Montreal Cognitive Assessment [n=23] | Geriatric Depression Scale [n=23] | Epworth Sleepiness Scale [n=23] |
| --- | --- | --- | --- | --- |
| Total Cortex | r=-0.137, p=0.533 | r=-0.089, p=0.686 | r=-0.218, p=0.318 | r=0.138, p=0.530 |
| Caudate & Putamen | r=-0.424, p=0.044 | r=0.086, p=0.697 | r=-0.069, p=0.753 | r=0.271, p=0.210 |
| Thalamus | r=-0.250, p=0.251 | r=-0.034, p=0.879 | r=-0.100, p=0.650 | r=0.354 p=0.097 |
| Limbic Cortex | r=-0.137, p=0.533 | r=0.013, p=0.953 | r=-0.221, p=0.310 | r=0.163 p=0.456 |
| Ventral Striatum | r=-0.236 p=0.279 | r=0.149 p=0.499 | r=-0.048 p=0.828 | r=0.382 p=0.073 |
| Ventral Anterior Cingulate | r=-0.234, p=0.282 | r=0.050, p=0.822 | r=-0.261, p=0.229 | r=0.140, p=0.524 |
| Substantia Nigra | r=-0.300, p=0.165 | r= 0.094, p=0.670 | r=0.121, p=0.583 | r=0.586, p=0.003 |
| Dorsal Raphe | r=-0.261, p=0.229 | r=-0.041, p=0.854 | r=0.125, p=0.570 | r=0.452, p=0.030 |
| Raphe Pontis | r=-0.229, p=0.292 | r=0.120, p=0.586 | r= 0.011, p=0.962 | r=0.338, p=0.115 |
| Medulla | r=-0.097, p=0.660 | r=0.041, p=0.854 | r=-0.296, p=0.170 | r=0.287, p=0.184 |

Supplementary Table 2: Bivariate Pearson’s correlations (r and p-value) between regional DASB distribution volume ratio and clinical scales in subjects with Multiple System Atrophy

|  | MDS-UPDRS Motor exam [n=14] | Montreal Cognitive Assessment [n=15] | Geriatric Depression Scale [n=14] | Epworth Sleepiness Scale [ n=14] |
| --- | --- | --- | --- | --- |
| Total Cortex | r=-0.670, p=0.009 | r= 0.196, p=0.485 | r=-0.450, p=0.111 | r=-0.269, p=0.353 |
| Caudate & Putamen | r=-0.682, p=0.007 | r=0.213, p=0.445 | r=-0.429, p=0.126 | r=-0.124, p=0.673 |
| Thalamus | r=-0.527, p=0.053 | r=0.056, p=0.842 | r=-0.177, p=0.546 | r=-0.083, p=0.777 |
| Limbic Cortex | r=-0.593 p=0.026 | r=0.063 p=0.823 | r=-0.450 p=0.107 | r=-0.190 p=0.515 |
| Ventral Striatum | r=-0.774 p=0.001 | r=0.199 p=0.476 | r=-0.383 p=0.177 | r=-0.265 p=0.359 |
| Ventral Anterior Cingulate | r=-0.789 p=0.001 | r= 0.376 p=0.167 | r=-0.408 p=0.147 | r=-0.342 p=0.232 |
| Substantia Nigra | r=-0.484 p=0.080 | r=-0.029 p=0.919 | r=-0.356 p= 0.211 | r=-0.508 p=0.064 |
| Dorsal Raphe | r=-0.380 p=0.180 | r=-0.066 p=0.814 | r=-0.498 p=0.070 | r=-0.033 p=0.912 |
| Raphe Pontis | r=-0.564 p=0.036 | r=0.007 p=0.979 | r=-0.537 p= 0.048 | r=-0.364 p=0.200 |
| Medulla | r=-0.524 p= 0.055 | r=0.180 p=0.522 | r=-0.318 p=0.269 | r=-0.534 p=0.049 |

Supplementary Table 3: Comparison of Regional DASB DVR between Normal Controls vs. Parkinson disease and Normal Controls vs. Multiple System Atrophy

| Region of Interest | Parkinson disease (PD; n=23) | Multiple System Atrophy (MSA; n=18) | Normal Controls (NC; n=16) | NC vs. PD (t-test, p-value) | NC vs. MSA (t-test, p-value) |
| --- | --- | --- | --- | --- | --- |
| Total Cortex | 1.267 (0.130) | 1.232 (0.093) | 1.792 (0.136) | t=12.205, p <0.001 | t=14.148, p <0.001 |
| Caudate & Putamen | 2.232 (0.182) | 2.210 (0.192) | 2.007 (0.175) | t=3.858, p<0.001 | t=3.201, p=0.003 |
| Thalamus | 2.381 (0.209) | 2.187 (0.164) | 2.103 (0.236) | t=3.887, p<0.001 | t=1.213, p=0.234 |
| Limbic Cortex | 1.553 (0.130) | 1.454 (0.138) | 1.799 (0.144) | t=5.571, p<0.001 | t=7.135, p<0.001 |
| Ventral Striatum | 2.388 (0.194) | 2.205 (0.241) | 1.959 (0.178) | t=7.016, p<0.001 | t=3.349, p=0.002 |
| Ventral Anterior Cingulate | 1.384 (0.115) | 1.360 (0.117) | 1.650 (0.137) | t=6.583, p<0.001 | t=6.664, p<0.001 |
| Substantia Nigra | 2.416 (0.360) | 2.401 (0.346) | 1.614 (0.173) | t=8.249, p<0.001 | t=8.224, p<0.001 |
| Dorsal Raphe | 2.970 (0.342) | 2.912 (0.390) | 1.700 (0.139) | t=14.035, p<0.001 | t=11.758, p<0.001 |
| Raphe Pontis | 2.566 (0.297) | 2.241 (0.300) | 1.556 (0.253) | t=11.089, p<0.001 | t=7.157, p<0.001 |
| Medulla | 1.571 ( 0.168) | 1.364 (0.210) | 1.464 (0.189) | t=1.859, p=0.071 | t=1.443, p=0.159 |

*NC = Normal Control; MSA = Multiple System Atrophy; PD = Parkinson disease; absolute values of t-test/chi-square are presented.

Supplementary Figure 1

Scatter plots depicting the inverse relationship in MSA subjects (n=14) between MDS-UPDRS motor score and regional DASB distribution volume ratio (DVR) in the A) Total Cortex [top left] B) Caudate & Putamen [top right] C) Ventral Anterior Cingulate [bottom left] and D) Raphe Pontis [bottom right]
